# Angiotensin-I Converting Enzyme (ACE) Inhibitory and Anti-Hypertensive Effect of Protein Hydrolysate from *Actinopyga lecanora* (Sea Cucumber) in Rats

**DOI:** 10.3390/md14100176

**Published:** 2016-09-30

**Authors:** Mahdokht Sadegh Vishkaei, Afshin Ebrahimpour, Azizah Abdul-Hamid, Amin Ismail, Nazamid Saari

**Affiliations:** 1 Department of Food Science, Faculty of Food Science and Technology, Universiti Putra Malaysia, Serdang 43400, Selangor, Malaysia; mahdokht_vishkaiee@yahoo.com (M.S.V.); azizahah@upm.edu.my (A.A.-H.); 2 Department of Chemistry, Sam Houston State University, Huntsville, TX 77340, USA; afshin.ebrahimpour@gmail.com; 3 Department of Nutrition and Dietetics, Faculty of Medicine and Health Sciences, Universiti Putra Malaysia, Serdang 43400, Selangor, Malaysia; amin@medic.upm.edu.my

**Keywords:** sea cucumber, ACE inhibition, normotensive rats, hypertension

## Abstract

Food protein hydrolysates are known to exhibit angiotensin converting enzyme (ACE) inhibitory properties and can be used as a novel functional food for prevention of hypertension. This study evaluated the ACE inhibitory potentials of *Actinopyga lecanora* proteolysate (ALP) in vivo. The pre-fed rats with ALP at various doses (200, 400, 800 mg/kg body weight) exhibited a significant (*p* ≤ 0.05) suppression effect after inducing hypertension. To determine the optimum effective dose that will produce maximal reduction in blood pressure, ALP at three doses was fed to the rats after inducing hypertension. The results showed that the 800 mg/kg body weight dose significantly reduced blood pressure without noticeable negative physiological effect. In addition, there were no observable changes in the rats’ heart rate after oral administration of the ALP. It was concluded that *Actinopyga lecanora* proteolysate could potentially be used for the development of functional foods and nutraceuticals for prevention and treatment of hypertension.

## 1. Introduction

Hypertension, a disorder that is characterized by a constant rise in levels of blood pressure, is a common and critical chronic health problem recognized as the most important risk factor for cardiovascular disease (CVD) throughout the world [1,2,3,4]. The prevalence of hypertension has been extensively reported in different regions of the world [5]. Previous studies done in 2005 demonstrated that nearly 1 billion individuals, or >25% of the population of the world, had high blood pressure in 2000. This statistic is predicted to rise to 1.56 billion by 2025 [6]. Nowadays, anti-hypertensive medications namely, analapril and captopril marketed under the trade names Accupril, Capoten, Altace, Lotensin, Prinvil, Vasotec, Monoril, Zestril and other synthetic ACE inhibitors such as Ramipril and Lisinopril are applied extensively in the clinical therapy of hypertension and also heart failure in humans. These effective synthetic ACE inhibitors stabilize blood pressure. However, they can have adverse effects such as skin rashes, taste disturbances, cough, drug-drug interactions and high cost [7,8,9]. Therefore, the search for natural food based inhibitors for the prevention of hypertension is currently of great interest to consumers and functional foods could potentially serve this need.

Peptides originating from food proteins could be developed into nutraceuticals which are known as natural and safe alternatives to synthetic drugs for the prevention and treatment of hypertension.

The most well established mechanism, which is based on the blood-pressure-lowering effect, is the inhibitory properties of angiotensin-converting enzyme (ACE). ACE performs a crucial role in the blood pressure regulation in the renin angiotensin system. It is a hormone system that adjusts blood pressure as well as fluid balance, and performs an essential function in the pathophysiology of CVDs, such as hypertension and heart failure [10].

ACE inhibitors from protein hydrolysate sources can be obtained from various foods such as bovine casein [11], fermented foods [12], red algae [13], etc. However, only in vivo studies can confirm the ability of ALP candidates to reduce blood pressure and reveal their antihypertensive effects.

*Actinopyga lecanora*, a sea cucumber, known as stone fish with moderately high protein content, was investigated as a raw material for production of bioactive peptides [14]. *Actinopyga lecanora* belongs to the marine invertebrate of the phylum Echinoderm and the *Holothuroidea* class. It is known as a by-catch of the fishery industry, and is not typically consumed as a food in Malaysia. Previous studies reported ACE inhibitory effect of *Actinopyga lecanora* bromelain generated proteolysate in vitro [14]. Therefore, the current study aimed to investigate ACE inhibitory potential of *Actinopyga lecanora* proteolysate in vivo. The main goal of the work was to produce an ACE inhibitor proteolysate derived from marine source with desirable functional characteristics comparable to synthetic drugs for amelioration of hypertension.

## 2. Results and Discussion

### 2.1. Effect of Proteolysate on Normal Blood Pressure

**Figure 1:**
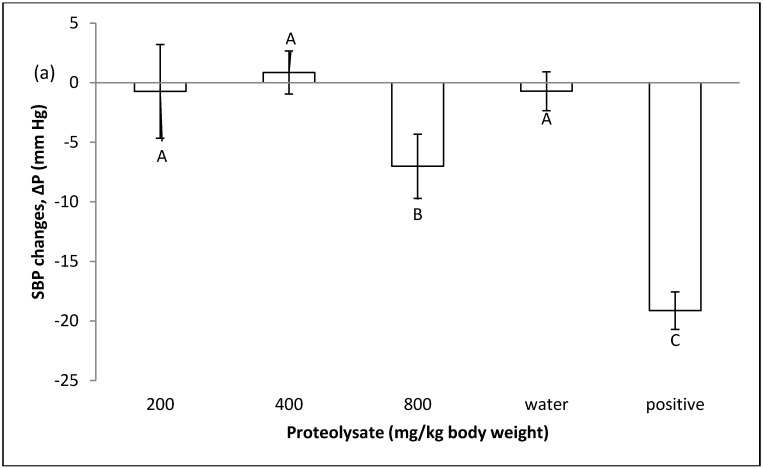

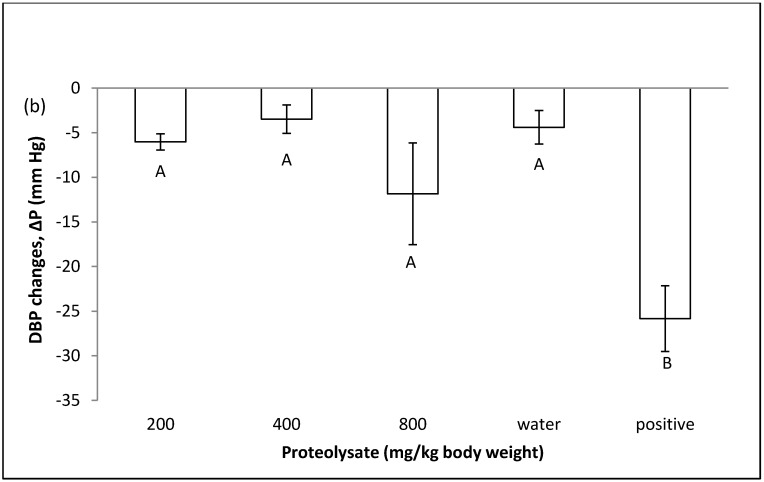
The effect of proteolysate (200, 400, 800 mg/kg body weight) on normal blood pressure. Positive group was given captopril (50 mg/kg body weight). Water control group was given distilled water. Δ*P* (mm Hg) shows blood pressure changes, which can be increase or decrease; (−) shows decrease and (+) shows increase in blood pressure. **A**–**C** significant differences at the confidence level of *p* ≤ 0.05 (mean ± SD, *N* = 5), (**a**) SBP; (**b**) DBP.

The effect of ALP on normal systolic and diastolic blood pressure was investigated by feeding normotensive rats at three different concentrations of 200, 400 and 800 mg/kg body weight and measuring blood pressure 1 h after oral administration. It can be seen in Figure 1 how the in vivo system responded to oral administration of the ALP. Rats receiving ALP at all 3 doses display significant differences with the synthetic ACE inhibitor (captopril), meaning that ALP at these doses did not affect normal blood pressure.

### 2.2. Preventive Group

#### 2.2.1. Changes in Blood Pressure in Pre-Fed Rats

**Figure 2:**
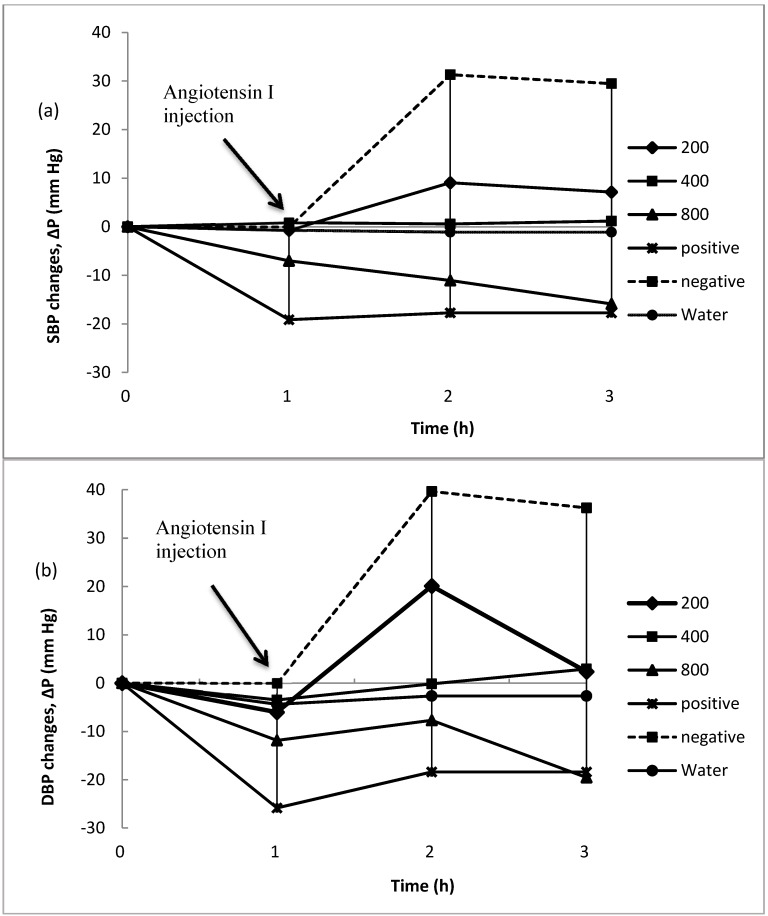
Δ*P* (mm Hg) showing blood pressure changes before and after inducing HBP via injections of angiotensin I (0.3 μg/kg body weight) and saline. Treated rats were given different doses of *Actinopyga lecanora* proteolysate (ALP) (200, 400, 800 mg/kg body weight) and positive group was given captopril (50 mg/kg body weight), (mean ± SD, *N* = 5), (**a**) SBP; (**b**) DBP.

The results of many previous studies demonstrate that hypertension might be affected by the ACE inhibitory peptides in protein hydrolysate depending on the type of peptides [15,16]. In order to study changes in blood pressure, normotensive rats were pre-fed with the ALP at three different concentrations of 200, 400 and 800 mg/kg body weight. Then 1 h after pre-feeding, they were injected angiotensin I to induce hypertension in them. Figure 2 displays the time-course changes in SBP and DBP after oral administration of distilled water, captopril and different concentrations of ALP. Before administration of different samples, the mean SBP and DBP changes of normotensive rats were zero. Rats SBPs and DBPs declined after oral administration of ALP at all concentrations; however, these SBP and DBP changes were not significant (*p* < 0.05). Systolic blood pressures (SBP) and diastolic blood pressure (DBP) increased significantly, by about 31 mm Hg and 40 mm Hg from baseline after angiotensin I was injected into the rats as a negative control, respectively. On the other hand, the SBP and DBP of rats decreased slightly, by approximately 18 mm Hg and 17 mm Hg from baseline after oral administration of captopril, respectively. Oral administration of various concentrations of ALP showed differing SBP and DBP changes. The oral administration of ALP at all concentrations showed hypotensive effect after inducing hypertension by injecting angiotensin I. Rat SBPs and DBPs increased after angiotensin I injection at a dose of 200 mg/kg body weight, indicating that the amount of hypotensive effect of ALP to suppress the rise in blood pressure was not adequate at this concentration. In contrast, rat SBPs and DBPs decreased at a dose of 800 mg/kg body weight after angiotensin I injection. The oral administration of ALP at a dose of 400 mg/kg bodyweight and distilled water showed no significant hypotensive effect and remained approximately constant during the period of the experiment.

#### 2.2.2. Effect of Proteolysates in the Prevention of Blood Pressure Increases after Inducing Hypertension

In this study, the preventive effect of ALP against hypertension was examined in vivo using normotensive rats [17,18,19,20]. This type of in vivo procedure is reported to be reliable in determining how effective a compound is in reducing or modulating blood pressure [19,20]. The modulating and hypotensive effect of ALP was evaluated at different concentrations using normotensive rats. This procedure was established by [17,18] and it was used with some modifications in the present study.

**Figure 3:**
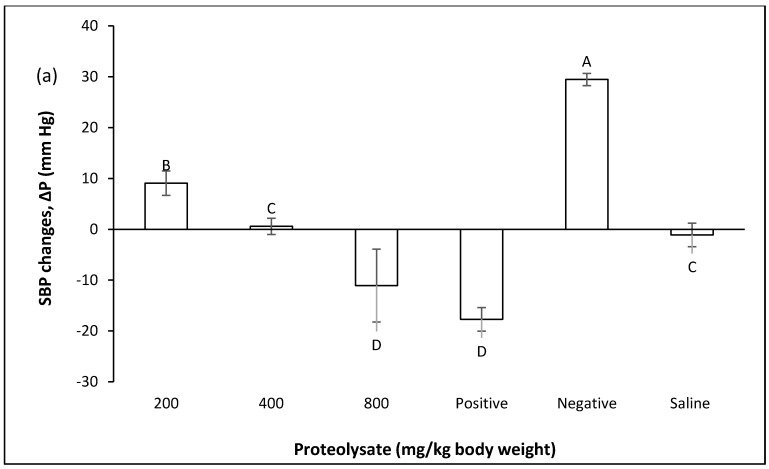

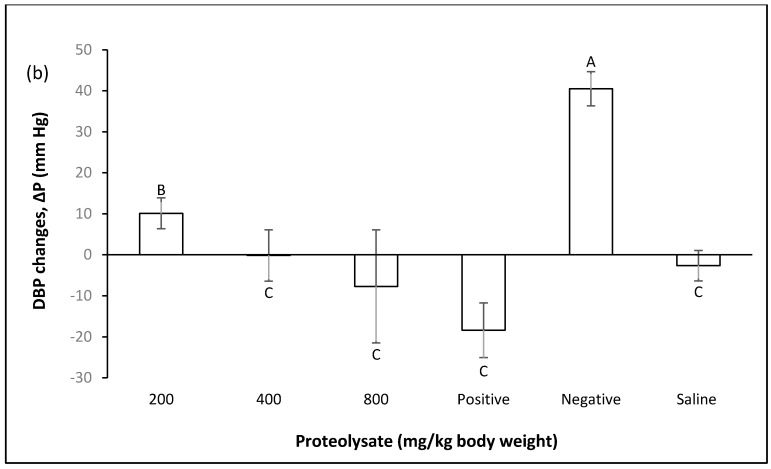
The effect of ALP at doses of 200, 400, 800 mg/kg body weight in pre-fed rats, after inducing hypertension by angiotensin I (0.3 μg/kg body weight) injection. The positive group was given captopril (50 mg/kg body weight). Δ*P* (mm Hg) shows blood pressure changes, which can be increase or decrease; (−) shows decrease and (+) shows increase in blood pressure. **A**–**D** significant differences at the confidence level of *p* ≤ 0.05 (mean ± SD, *N* = 5), (**a**) SBP; (**b**) DBP.

Generally, the results indicated that ALP possessed the ACE inhibitory effect at all three doses and showed hypotensive effect on both systolic blood pressure (SBP) and diastolic blood pressure (DBP) significantly in rats pre-fed with ALP. Despite the negative group was pre-fed with distilled water, the same result was observed for captopril as a positive group (Figure 3).

The SBPs and DBPs responses to angiotensin I in the water control group and negative control group are significantly different (*p* ≤ 0.05). It shows that the blood pressure was not affected by the experimental procedure (such as handling the rats, force-feeding and injections) and the statistically significant findings were not due to experimental procedures. Despite the results that were observed for SBP, significant differences were not observed between the positive group and water group in DBP. It reveals the fact that SBP could only be affected by a synthetic drug (captopril) in normotensive rats. In other words, the magnitude of DBP inhibition is quite low compared to SBP in the positive group. The same results were observed for doses of 400 and 800 mg/kg body weight in DBP. Significant differences in SBP were not detected at a dose of 800 mg/kg body weight and positive control. The results showed that a dose of 800 mg/kg body weight possesses the same magnitude inhibition as captopril. While similar results were discovered for a dose of 400 mg/kg body weight and water group in SBP and these findings lead us to conclude that ALP at this dose possesses an inhibitory effect and normal blood pressure is not affected by ALP. However, the same magnitude of inhibition was not observed compared to what can be achieved in positive control (captopril). Figure 3 shows that the inhibition of DBP can be seen at a dose of 200 mg/kg body weight which is not quite significant.

It had been previously reported that the pressor effect of angiotensin I (0.3 μg/kg) was significantly lower when rats were pre-fed with milk fermented using two strains of *Lactobacillus helveticus* [19]. However, ALP as a functional food at regular consumption or even lower concentration may give a positive accumulative blood pressure stabilizing effect. Moreover, the baseline blood pressure in the above-mentioned study has not been considered as such and the rats were unconscious and have been subjected to invasive surgery. The results of present study reveal that *A. lecanora* in general produce peptides, which are inhibitory toward ACE, however it seems that only a dose of 800 mg/kg body weight is large enough in amount to cause an effect directly on ACE in vivo.

### 2.3. Treatment Group

#### 2.3.1. Changes in Blood Pressure after Inducing Hypertension

**Figure 4:**
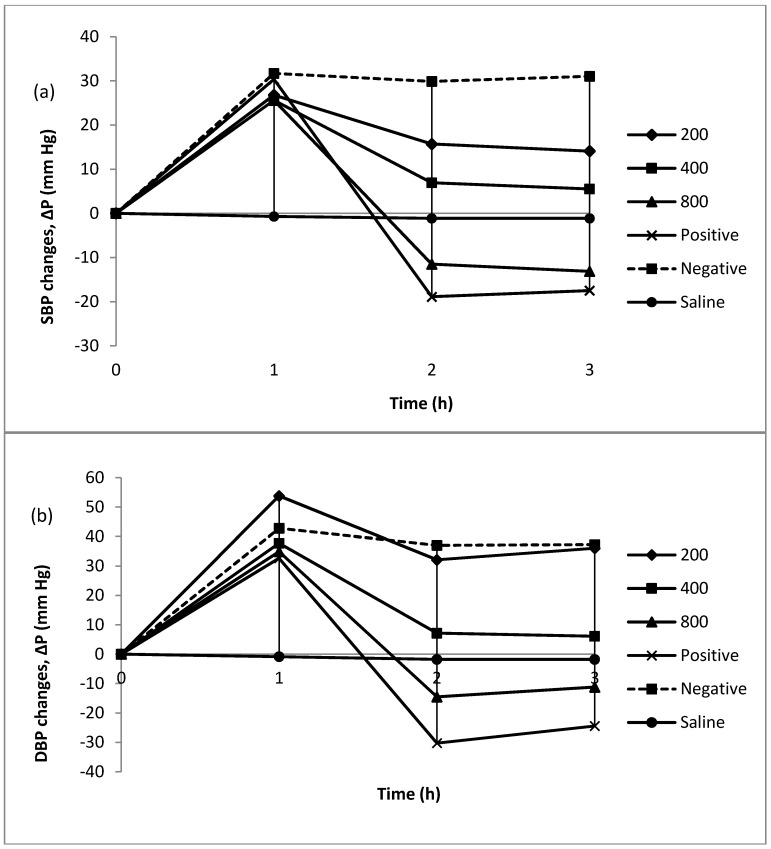
Δ*P* (mm Hg) showing blood pressure changes before and after inducing HBP via injections of angiotensin I (0.3 μg/kg body weight) and saline. Treated rats were given different doses of ALP (200, 400, 800 mg/kg body weight) and positive group was given captopril (50 mg/kg body weight), (mean ± SD, *N* = 5), (**a**) SBP; (**b**) DBP.

In order to study the curative potential of proteolysate as a blood pressure lowering drug, the present research was carried out by inducing hypertension before force feeding the ALP. Figure 4. depicts time-course changes in systolic blood pressure (SBP) and diastolic blood pressure (DBP) after inducing hypertension with angiotensin I and force-feeding ALP at different concentrations. It can be seen that inducing hypertension with injection of angiotensin I was successful. SBP and DBP reached a peak 1 h after injection in all groups, although the water group remained constant during the period of experiment. 1 h after force-feeding ALP, there were sharp drops in SBP at a dose of 800 mg/kg body weight and positive control group of approximately 13 mm Hg and 18 mm Hg, respectively. On the other hand there was a moderate decrease of nearly 7 mm Hg in SBP at a dose of 400 mg/kg body weight and a slight decline of around 14 mm Hg at a dose of 200 mg/kg body weight while the negative control group reached a plateau. It can be seen DBP also exhibited the same pattern as SBP. However, DBP at a dose of 200 mg/kg body weight was not decreased considerably compared to SBP.

Many studies have been conducted to investigate the short-term antihypertensive effects of bioactive peptides that come from food proteins [9,21,22]. It has been shown that food protein peptides are bioavailable and can exert effects in a relatively short term [21,23,24,25,26,27]. Among food sources, there is huge source of protein in seafood coming from ocean because oceans contain diverse marine organisms. In previous study the anti-hypertensive activity of jelly fish (*Rhopilema esculentum*) protein hydrolysate which was hydrolyzed with pepsin and papain in both acute and chronic study were investigated [15]. SBP in spontaneously hypertensive rats was lowered significantly after single oral administration of protein hydrolysate at doses of 200, 400 and 800 mg/kg body weight with maximal fall of 23.00 mm Hg, 27.34 mm Hg and 30.41 mm Hg, respectively. Although normotensive rats were used in the present study, administration of doses 200, 400 and 800 mg/kg body weight led to maximal decrease of systolic blood pressure by 15.67 mm Hg, 24.33 mm Hg, 45.00 mm Hg, respectively. These normotensive rats were subjected to hypertension via angiotensin I injection. In current investigation, the maximal fall was within dose of 800 mg/kg body weight which was more than previous one due to higher ACE inhibitory activity of ALP that hydrolyzed with bromelain. The amount of reduction at a dose of 800 mg/kg body weight ALP was approximately similar to the amount of reduction in the chronic study of above-mentioned one at doses of 400 and 800 mg/kg body weight which constitutes for 46.90 mm Hg and 43.21 mm Hg, respectively [15]. Matsui et al. [28] indicated that a vegetable drink containing sardine protein hydrolysate that exhibited in vitro ACE inhibitory activity showed the antihypertensive effect exclusively in the subjects with high-normal blood pressure or mild hypertension; while no adverse effects were observed in either normotensive or hypertensive subjects. The same outcomes were also reported with thermolysin digest of dried bonito, named “Katsuobushi oligopeptide”, which has peptide with ACE inhibitory effect. Ministry of Health and Welfare in Japan officially approved this product as Foods for specified health use [9]. In the present study, the SBP lowering effect of ALP was less than that of captopril in different dose groups, in the acute study. These features were also confirmed in other earlier studies [9,11,29]. In non-marine sources, similar researches were carried out. Li et al. [9], examined the anti-hypertensive effect of rice protein hydrolysate prepared with Alcalase at a dose of 600 mg/kg body weight. They reported that rice protein hydrolysate at this dose indicated a significant decrease in SBP with maximal fall of 25.6 mm Hg.

#### 2.3.2. Curative Potential of Proteolysate

**Figure 5:**
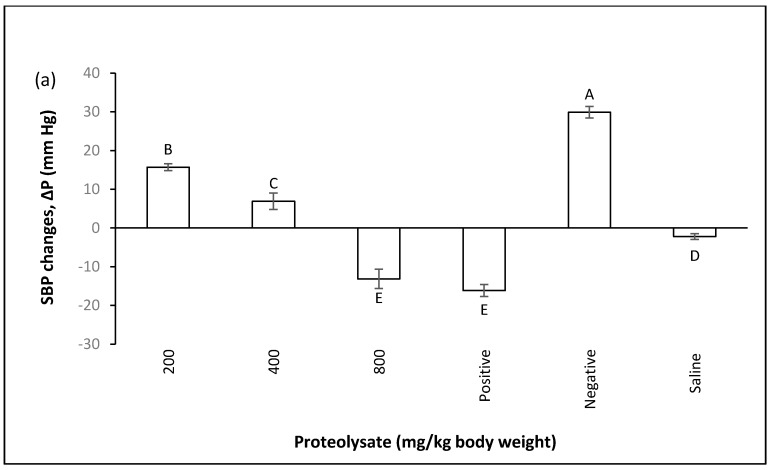

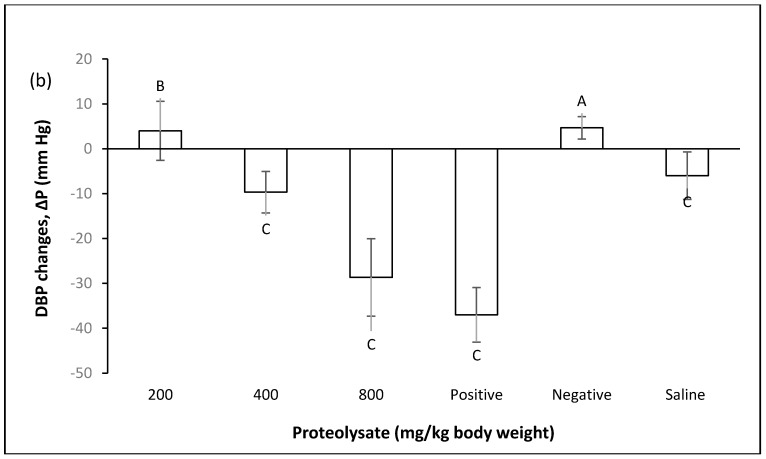
The effect of ALP at doses of 200, 400, 800 mg/kg body weight after inducing hypertension by angiotensin I (0.3 μg/kg body weight) injection. The positive group was given captopril (50 mg/kg body weight). Δ*P* (mm Hg) shows blood pressure changes, which can be increase or decrease; (−) shows decrease and (+) shows increase in blood pressure. **A**–**D** significant differences at the confidence level of *p* ≤ 0.05 (mean ± SD, *N* = 5), (**a**) SBP, (**b**) DBP.

Figure 5 displays that high SBP and DBP induced by angiotensin I were decreased by ALP at all three doses. It was shown that there were significant differences between water control group and negative control group, which means that statistically significant changes were not due to experimental procedure. Significant differences were not observed between synthetic drug and ALP at a dose of 800 mg/kg body weight. Thus, it can be concluded that ALP at a dose of 800 mg/kg body weight possesses the same curative potential as captopril. However, the amounts of reduction in other two doses are lower compared to what can be reached with captopril. The low level of reduction shows that ALP at these doses do not possess the same range of treatment potential as a drug. The same pattern was observed for DBP. However, in contrast to SBP, doses of 200 and 400 mg/kg body weight did not exert significant reduction in DBP while a dose of 800 mg/kg body weight decreased DBP remarkably and possesses the same curative potential as captopril. Significant differences were not observed between a dose of 800 mg/kg body weight and water control group. Thus, it can be concluded that it can reach almost the baseline after reduction of the DBP. The same results were reported by Xu Wang [30]. They investigated the antihypertensive effect and ACE inhibitory activity of whey protein hydrolysate after single oral administration (short term) on spontaneously hypertensive rats. Whey protein hydrolysate at three doses of 80, 240 or 1200 mg/kg body weight reduced the SBP and DBP of animals. In the above -mentioned study, which is similar to the present study, both SBP and DBP show significant reduction after orally taking whey protein hydrolysate and captopril. In fact, their blood pressures were lower than that of the negative control group (*p* ≤ 0.05) and SBP was markedly lower in the captopril groups than that of the protein hydrolysate groups (*p* ≤ 0.05). It truly signifies that captopril had more effective anti-hypertensive activity than whey protein hydrolysate in Xu Wang study and *Actinopyga lecanora* proteolysate in present study [30].

### 2.4. Heart Rate

Heart rate can be an independent risk factor in individuals with hypertension for cardiovascular death [30,31]. There is a substantial rise in consumption of oxygen per beat as heart rate increases. The increased consumption of myocardial oxygen due to increased heart rate results in increased myocardial load [30,32]. This study checked the effects of different doses of proteolysate on the heart rate and the physical condition after one oral administration of the hydrolysate. There were no significant changes in the heart rate of animals in all groups after oral administration, thus leading to the conclusion that administering the hydrolysate had no negative effect on the circulatory system of rats in both preventive and treatment group (Table 1). The same result was also reported in earlier studies in 2007 by Li et al. [9] and in 2012 by Liu et al. [15].

## 3. Materials and Methods

### 3.1. Material

The fresh samples (*Actinopyga lecanora*) were purchased from a local supplier in Pantai Merdeka, Malaysia. After removing internal organs, samples were washed, packed and kept at −80 °C for further use. Angiotensin I (Sigma-Aldrich, Kuala Lumpur, Malaysia, US/A9650) and captopril (Sigma-Aldrich, Kuala Lumpur, Malaysia, US/PHR1307) were purchased from Next Gene Scientific Sdn Bhd. Bromelain was obtained from Acros Organics Co. (St. Louis, MO, USA).

### 3.2. Animals

Sprague dawley rats (white, male, 10–15 weeks, 250 ± 10 g body weight, specific pathogen-free) were purchased from Sapphire Enterprise supplier in Malaysia and acclimatized for 10 days at room temperature (24 ± 1 °C), humidity of 50% ± 10%, and a 12-h dark/12-h light cycle. During the acclimatization period, all the rats had free access to laboratory chow and distilled water. All of animal care and maintenance procedures were approved by the Institutional Animal Care and Use Committee (IACUC) of the Faculty of Veterinary Medicine, Universiti Putra Malaysia (Ref: UPM/IACUC/AUP-R050/2013, date: 30/1/2014).

### 3.3. Preparation of Proteolysate

The samples were freeze-dried and ground with a waring blender and then sieved (mesh size 600 micro meter). The powdered sample was kept at −80 °C until they were used. Hydrolysis was carried out according to the method of a previous study [14]. Powdered sample (10 g) was mixed with 50 mL distilled water and dialyzed in a dialysis tubing with 12–14 kDa molecular weight cut off (MWCO) (Dialysis Tubing-Visking, 28.6 mm diameter). The tube was closed tightly at both ends and immersed in an appropriate buffer solution (50 mM) for 24 h at 4 °C. After dialysis, the sample was incubated in a water bath to reach the reaction optimum temperature for bromelain enzyme. Bromelain was dissolved in an appropriate buffer solution, and mixed well with dialyzed sample in the test container. The enzyme/substrate ratio was adjusted to 1/100 (*w*/*w*) based on the protein content of *A. lecanora*. Proteolysis was carried out for 1 h in a water bath with continuous stirring at 150 rpm under the optimum conditions for bromelain enzyme; acetate buffer (50 mM), pH 5.5, temperature 55 °C and agitation rate 150 rpm. The enzymatic reaction was immediately terminated by heating the samples in a boiling water bath for 15 min to inactivate the proteases. The proteolysates were rapidly cooled down in an ice bath and then centrifuged at 10,000× *g*, for 20 min at 4 °C (Sigma, Kuala Lumpur, Malaysia, 3–18 k) using a refrigerated high-speed centrifuge to remove insoluble materials. The resulting supernatant containing peptides was collected, and stored at −80 °C before being freeze-dried using freeze-dryer (Scanvac-Collsafe 110-4, LaboGene, Lynge, Denmark).

### 3.4. In Vivo Study

The powder (freeze-dried) proteolysate (200 mg/kg, 400 mg/kg, 800 mg/kg) dissolved in 2.0 mL distilled water and were administered by oral gavage. Distilled water (2.0 mL) and angiotensin I (0.3 μg/kg) served as the negative control groups, and captopril (50 mg/kg), a well-known ACE inhibitor, served as the positive control. The methods of Liu et al. [15] and Fuglsang et al. [17] had been used with some modifications based on preliminary study [15,17]. Systolic blood pressure and diastolic blood pressure of pre-warmed conscious rats were measured by tail cuff method using CODA machine (No. 20846, Non-invasive volume pressure recording, Kent Scientific Corporation, San Diego, CA, USA). The heart rate was measured at the same time. Prior to measurement, rats were kept at 37 °C for 10 min to make pulsation of the tail artery detectable. To minimize interference by peptides that might arise from digestion of protein in the feed of animal, rats were fasted for 6 h prior to proteolysate oral administration. However, rats were allowed to drink water throughout the fasting period. The doses described by Liu et al. [15] were used in this study. Systolic blood pressure (SBP) and diastolic blood pressure (DBP) of the rats were measured by the tail-cuff method before administration. And they were again measured 1, 2 and 3 h after administration using a tail-cuff apparatus. Fifteen readings at minimum were also recorded. The maximum and minimum values were rejected, and the blood pressure was calculated as the average of the remaining values [15,17].

### 3.5. Effect of Proteolysate on ACE in Preventive Group

Blood pressure was determined 1 h after proteolysate administration to observe the effect of proteolysate on initial blood pressure [15]. Then Angiotensin I at a dose of 0.3 μg/kg body weight was injected to induce high BP using intravenous injection [17]. For intravenous injection, conscious rats were restrained in the animal holder and tail vein were used. Animal holder was selected carefully to be an appropriate size for the animal to minimize the tail movement. In positive control group, captopril, a well-known ACE inhibitor, was orally administered at a dose of 50 mg/kg after initial BP measurement. The same process was applied for treated groups. In Angiotensin negative group, Angiotensin I in the same dose was injected after initial BP measurement, and BP was measured every 1 h after injection to ensure of induction of high blood pressure (HBP) and keeping BP at a high level during the time of the whole process. In water negative group, the whole procedure was simulated with force feeding of distilled water and injection of saline in order to observe the effect of handling the rats, force feeding, injection and the stress arising from BP changes in the rats.

### 3.6. Effect of Proteolysate on High Blood Pressure in Treatment Group

New groups of rats were used for this study. After initial BP measurement, Angiotensin I at a dose of 0.3 μg/kg body weight was injected to induce HBP using intravenous injection method. 1 h after injection, BP of rats were measured to be sure of inducing HBP. Then the proteolysate, which was dissolved in 2 mL distilled water, was orally administered at 3 doses (200, 400, 800 mg/kg body weight). In positive control group Angiotensin I (0.3 μg/kg body weight) was injected after initial BP measurement to induce hypertension using intravenous injection. Captopril (50 mg/kg body weight) was administered to decrease HBP. In Angiotensin negative group, after initial BP measurement, Angiotensin I in the same dose was injected and BP was measured every 1 h after injection to be sure of inducing HBP and keeping BP at a high level during the time of the whole process. In water negative group, the whole procedure was simulated with injection of saline and force feeding of distilled water, respectively in order to observe the effect of handling the rats, force feeding, and injection on rats BP.

### 3.7. Data Analysis

All statistical analysis were performed using Minitab version 16.0 (Minitab Inc., State College, PA, USA) and the differences between treated and control groups were assessed by one way analysis of variance (ANOVA) at a confidence level of 95% and *p* values less than 0.05 were considered significant. All data are reported as means ± standard deviations (SD).

## 4. Conclusions

Since hypertension is becoming a serious health issue all over the world, lots of studies have been done about ACE inhibitory peptides, which are derived from food protein. There has been evidence of their antihypertensive effect in vivo, in both clinical and animal studies. The present study is the first in which an in vivo effect of *A. lecanora* on ACE has been reported. The ultimate goal of this study was to investigate ACE inhibitory properties of proteolysate derived from marine source, which can be used as alternative therapy for prevention and treatment of hypertension. The results presented lead to the conclusion that *A. lecanora* can be used as a raw material for the bioactive peptides generation because of its relatively high protein content. *A. lecanora* generally produce peptides, which have ACE inhibitory effect in vivo, however, it seems that only a dose of 800 mg/kg body weight which are large enough in amounts to show obvious effect on ACE. The current work showed that ALP at all three doses had no effect on normal systolic and diastolic blood pressure, significantly. The investigation of the effect of different doses on heart rate displayed no significant differences. It can lead us to conclude that administering the proteolysate had no negative effect on the circulatory system of rats in both preventive and treatment groups.

The focus of current study was on the examination of the acute blood pressure-lowering effects of *Actinopyga lecanora* proteolysate by oral administration. More studies are certainly needed to scrutinize the effects of dose-response of the long-term consumption of these products and establish the minimum effective dose value on the progress of hypertension in animal models. In addition, future studies should focus on investigation of the effects of the *A. lecanora* in spontaneously hypertensive rats. As proteolysate had no effect on normal blood pressure, it is recommended to incorporate the bioactive components derived from *A. lecanora* into food and drink products and develop functional foods and drinks with hypotensive benefits. As such, consumer acceptability is of utmost importance. Since peptides released from food protein via enzymatic hydrolysis tend to give bitter taste, sensory evaluation is recommended for such products; because when sensory characteristics do not fit expectations, products will not be welcomed.

## Figures and Tables

**Table 1:** Heart rate of Sprague dawley (SD) rats after administration of the proteolysate before and after inducing hypertension.

Group	mg/kg Body Weight	HR Preventive Group (BPM)	HR Treatment Group (BPM)
Proteolysate concentration	200	363.06 ± 25.51	333.00 ± 6.56
Proteolysate concentration	400	368.00 ± 30.81	365.33 ± 18.90
Proteolysate concentration	800	321.50 ± 90.06	358.67 ± 10.41
Positive control	50	339.33 ± 42.34	372.67 ± 23.03
Negative control (AngiotensinI)	-	332.42 ± 59.88	380.75 ± 10.63
Negative control (Water)	-	382.00 ± 10.15	338.67 ± 77.23

Values are means ± SD for 5 rats/Group. The differences between the control and treated groups were evaluated by one way ANOVA. No significant differences at the confidence level of *p* ≤ 0.05 were observed.

## Abbreviation

ACE	Angiotensin converting enzyme
SBP	Systolic blood pressure
DBP	Diastolic blood pressure
HR	Heart rate
CVD	Cardiovascular disease
SD	Sprague dawley
